# Pericardio-diaphragmatic rupture after blunt trauma: a case report

**DOI:** 10.3389/fsurg.2025.1693409

**Published:** 2025-10-21

**Authors:** Dayi Xing, Boyu Xia, Jiandong Yang, Yuansheng Zhao

**Affiliations:** 1 Department of Cardiothoracic Surgery, Sinopharm Tongmei General Hospital, Datong, Shanxi, China; 2 Department of Cardiothoracic Surgery, Affiliated Hospital of Nantong University, Nantong, Jiangsu, China

**Keywords:** blunt trauma, traumatic diaphragmatic rupture, intrapericardial hernia, sternotomy, thoracoscopy

## Abstract

**Background:**

Pericardio-diaphragmatic rupture with intrapericardial herniation is a rare and potentially life-threatening complication of blunt thoracoabdominal trauma. Its diagnosis is challenging because pericardial involvement is often missed on imaging.

**Case presentation:**

We present the case of a 70-year-old man who sustained blunt chest trauma in a motor vehicle collision. On admission, he was hemodynamically stable, and echocardiography demonstrated preserved left ventricular function (ejection fraction 59%) without pericardial effusion. Initial CT demonstrated multiple right rib fractures and pulmonary contusion. Repeat CT at our center revealed bilateral lower lobe atelectasis, small pleural effusions, and a bowel gas shadow anterior to the heart, suggestive of diaphragmatic rupture with intrapericardial herniation. Thoracoscopic exploration excluded right-sided injury; however, laparoscopic inspection identified a large left diaphragmatic tear (10 cm) with bowel and omentum herniating into the pericardial sac in direct contact with the epicardial surface. Due to limited exposure and high tension, the procedure was converted to median sternotomy for safe repair. Postoperative CT confirmed resolution of the hernia. The patient recovered uneventfully and remained asymptomatic at 3-month follow-up.

**Conclusions:**

Pericardio-diaphragmatic rupture with intrapericardial herniation is rare and often underdiagnosed because of nonspecific clinical features and subtle imaging findings. Median sternotomy should be considered when minimally invasive repair is not feasible, and combined thoracoabdominal evaluation is crucial for diagnosis and management.

## Introduction

Traumatic diaphragmatic rupture occurs in 0%–5% of patients with major blunt thoracoabdominal trauma. Pericardial rupture is even rarer, with an incidence of 0.4%–3.3% in traumatic diaphragmatic rupture cases (1). The condition is most often left-sided due to the protective effect of the liver on the right hemidiaphragm. When diaphragmatic and pericardial ruptures occur together, abdominal viscera may herniate into the pericardial sac, resulting in pericardio-diaphragmatic rupture (PDR) (2). Diagnosis remains difficult: CT may show indirect signs such as abnormal bowel gas or mediastinal shift, but pericardial tears are frequently overlooked (3). Delayed presentations have been reported, sometimes days to months after the trauma, due to progressive enlargement of initially small defects (2, 3). To date, fewer than 20 cases of pericardio-diaphragmatic rupture with intrapericardial herniation have been reported in the literature, highlighting the rarity and clinical significance of this condition. Here, we describe a rare acute case of PDR with intrapericardial herniation in an elderly man, highlighting diagnostic pitfalls and operative strategies.

## Case presentation

A 70-year-old man presented to the emergency department 5 h after a motor vehicle collision, complaining of right-sided chest pain and dyspnea. On admission, his vital signs were: blood pressure 132/78 mmHg, heart rate 95 beats per minute, respiratory rate 22 breaths per minute, and oxygen saturation 96% on room air. He remained hemodynamically stable during the initial evaluation. Transthoracic echocardiography demonstrated preserved left ventricular systolic function (ejection fraction 59%) without pericardial effusion or tamponade.

**Figure 1:**
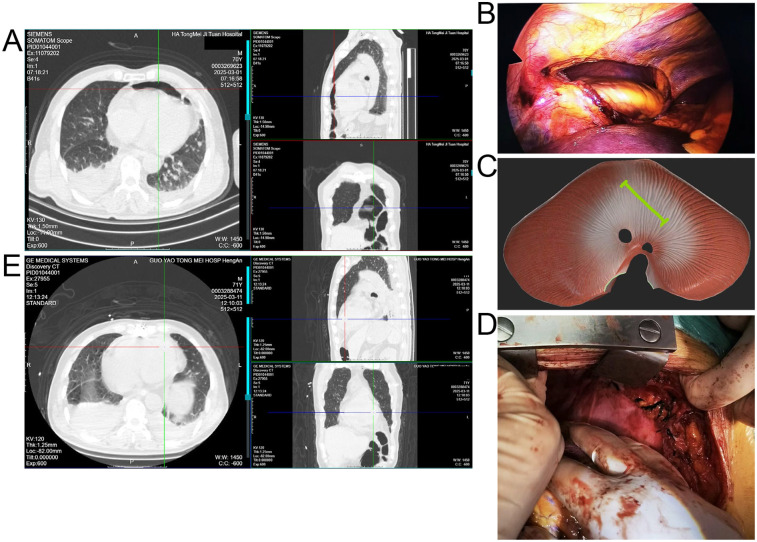
**(A)** Preoperative CT showing right rib fractures, bilateral lower lobe atelectasis, pleural effusions, and bowel shadow anterior to the heart suggestive of diaphragmatic hernia. **(B)** Laparoscopic view of herniated bowel and omentum entering the pericardial sac through a left diaphragmatic defect. **(C)** Schematic illustration of the diaphragmatic tear (green line) and intrapericardial herniation. **(D)** Intraoperative image after reduction and pericardial repair via median sternotomy. **(E)** Postoperative CT confirming resolution of the hernia and lung re-expansion.

At an outside hospital, CT showed multiple right rib fractures and pulmonary contusion. Upon transfer to our center, repeat CT revealed bilateral lower lobe atelectasis, small pleural effusions, and a bowel gas shadow anterior to the heart, raising concern for diaphragmatic rupture with intrapericardial herniation (Figure 1A). Thoracoscopic exploration demonstrated no right-sided diaphragmatic or pericardial rupture, which had been suspected based on the outside CT. Laparoscopy was subsequently performed to allow a more comprehensive evaluation of the left hemidiaphragm, and it revealed a 10 cm tear with herniation of bowel and omentum into the pericardial sac (Figure 1B,C). The herniated contents were in direct contact with the epicardial surface of the heart. Because of the large size of the defect and the high tension on the incarcerated viscera, safe reduction and repair could not be achieved under minimally invasive conditions. Therefore, the procedure was converted to a median sternotomy, which provided optimal exposure for reduction of the herniated contents, suspension of the pericardium, and secure closure of the defect (Figure 1D).

Postoperative CT confirmed resolution of the hernia and re-expansion of the lungs (Figure 1E). The patient was monitored in the ICU for 48 h and then transferred to the general ward. He experienced no major postoperative complications and was discharged on postoperative day 12 in good condition. At 3-month follow-up, he remained asymptomatic, and imaging confirmed sustained resolution of the hernia.

## Discussion

Pericardio-diaphragmatic rupture represents a spectrum of injuries that includes diaphragmatic lacerations with extension into the pericardium, avulsion of the pericardial sac from the central tendon, and combined defects at congenitally fused sites (4, 5). In adults, it is usually due to blunt trauma, particularly high-energy motor vehicle collisions (3). Although congenital weakness at the central tendon–pericardium junction has been implicated in some avulsion injuries, most cases result from sudden elevations in intra-abdominal pressure transmitting through the diaphragm (1).

The presence of pericardial involvement greatly increases diagnostic difficulty: while large diaphragmatic ruptures with herniated abdominal viscera are usually visible on CT, pericardial rupture alone may remain occult and often requires intraoperative confirmation (5). In our patient, CT was chosen as the primary diagnostic tool because of its accessibility and ability to rapidly evaluate both thoracic and abdominal injuries in the acute trauma setting. MRI was not considered due to the need for urgent surgical management. Furthermore, pericardio-diaphragmatic rupture is frequently underdiagnosed because the clinical presentation is often nonspecific and CT findings may be subtle. Indirect signs such as a bowel gas shadow anterior to the heart, as in our case, can easily be overlooked or misinterpreted. In addition, small pericardial tears without obvious herniation may not be detected until surgical exploration. These imaging pitfalls and clinical challenges highlight the need for a high index of suspicion in patients with blunt thoracoabdominal trauma. Delayed recognition has been reported, with patients presenting with tamponade, arrhythmias, or bowel obstruction weeks to months after trauma (3).

Surgical repair is mandatory once PDR is identified to prevent fatal complications such as cardiac herniation, tamponade, or strangulation of abdominal contents (1). Minimally invasive techniques can aid diagnosis and repair in stable patients, but conversion to open procedures is frequently required for large or high-tension defects, as in our patient (6). Alternative approaches such as laparotomy and thoracotomy have also been described. Laparotomy may be suitable for isolated diaphragmatic injuries but does not provide adequate exposure for intrapericardial herniation or pericardial repair. Thoracotomy allows direct access to the pericardium but limits visualization of abdominal viscera. In our case, the large size of the defect and the high tension on the herniated contents made minimally invasive repair unsafe. Therefore, median sternotomy was selected as the most appropriate strategy, as it offered wide exposure, enabled safe reduction of the herniated viscera, and allowed secure closure of the combined diaphragmatic–pericardial defect.

Some reports suggest that large pericardial defects may be intentionally left open to avoid constrictive complications, especially when the risk of cardiac incarceration is low (1). Nevertheless, primary repair is often feasible and recommended when the defect is smaller and tension-free closure can be achieved (1). Clinicians should be alert to indirect imaging signs such as bowel gas shadows anterior to the heart and unexplained mediastinal shift, especially in patients with high-energy blunt trauma. Persistent chest pain or dyspnea out of proportion to radiographic findings should also raise suspicion for pericardial involvement. Our case underscores the importance of maintaining suspicion for pericardial involvement in diaphragmatic injuries and supports combined thoracoabdominal surgical evaluation for definitive management (4, 7).

## Data Availability

The original contributions presented in the study are included in the article/Supplementary Material, further inquiries can be directed to the corresponding author.
